# Genome Sequence of *Pectobacterium carotovorum* Phage PPWS1, Isolated from Japanese Horseradish [*Eutrema japonicum* (Miq.) Koidz] Showing Soft-Rot Symptoms

**DOI:** 10.1128/genomeA.01625-15

**Published:** 2016-04-21

**Authors:** Hisae Hirata, Misako Kashihara, Tokumasa Horiike, Tomohiro Suzuki, Hideo Dohra, Osamu Netsu, Shinji Tsuyumu

**Affiliations:** a Department of Agriculture, Graduate School of Integrated Science and Technology, Shizuoka University, Suruga-ku, Shizuoka, Japan; b Research Institute of Green Science and Technology, Shizuoka University, Suruga-ku, Shizuoka, Japan

## Abstract

*Pectobacterium carotovorum* subsp. *carotovorum* and its lytic bacteriophage PPWS1 were isolated from a Japanese horseradish rhizome with soft rot. Sequencing of the phage genomic DNA suggested that PPWS1 is a new species of the family *Podoviridae* and has high similarity to the bacteriophage Peat1 infectious to *P. atrosepticum*.

## GENOME ANNOUNCEMENT

Soft rot of Japanese horseradish (wasabi) is a seasonally serious disease caused by *Pectobacterium carotovorum* subsp. *carotovorum* (PCC), *P. carotovorum* subsp. *wasabiae*, or some other soft rot bacteria (1–3). For preventing bacterial disease, phage therapy could be useful, especially in a wasabi field where agricultural chemicals cannot be applied. A lytic phage infectious to PCC isolated from soft-rotted wasabi was found in a plaque assay of a water sample of smashed wasabi rhizome tissue, and named PPWS1 (*Pectobacterium* phage isolated from wasabi cultivated in Shizuoka).

Genomic DNA of PPWS1 was purified using a high pure viral nucleic acid kit (Roche), then fragmented by Covaris shearing. A paired-end library constructed using the TruSeq Nano DNA Sample prep kit (Illumina) was sequenced using the Illumina MiSeq platform (2 × 301 bp). The raw reads (39,316 paired reads) were cleaned up with cutadapt (4) by trimming adapter sequences and low-quality ends (quality score, <30), resulting in 31,024 paired-reads totaling approximately 15.5 Mb. The cleaned reads were *de novo* assembled using SPAdes (5). The k-mer coverage of the assembled single contig was analyzed using JELLYFISH (6) to determine the terminal sequence; the length of the PPWS1 genome is 44,539 bp containing 247 bp of direct terminal repeats. An average 348× coverage of the total length was confirmed, and G+C content was 51.06%.

In a gene prediction and annotation using Prokka (7), 55 open reading frames (ORFs) were found. In a search for homologs in the NCBI nonredundant (nr) database using BLASTp, 31 of the ORFs had predicted functions and 24 were hypothetical proteins. Most ORFs showed high similarity to those of *P. atrosepticum* phage Peat1 (8), a newly reported member of *Podoviridae*. Morphological analysis revealed short-tailed particles, suggesting that PPWS1 is a member of the *Podoviridae*. Phylogenetic analysis based on putative RNA polymerase, the capsid protein and head-tail connector protein showed the closest relationship with Peat1, and they clustered with phages belonging to the genus *Phikmvlikevirus* and with phage KP34 (9). The typical genome architecture is preserved among most phikmvlikeviruses and KP34, that is, the last ORF in the early and middle regions is predicted to be a DNA-dependent RNA polymerase (10, 11), which PPWS1 also shares. Based on features of its genome and gene contents, KP34 is now the type species of the newly proposed genus *Kp34likevirus*, which includes some *Klebsiella pneumoniae* phages (12). The gene order in the lysis cassette (12) resembles that of Kp34, not that of phiKMV, while phylogenetic analysis showed a closer relationship to phikmvlikeviruses rather than kp34likeviruses. With sufficient genomic information for homologous phages, the genera of PPWS1 can be defined.

Factors conserved among PCC phages that determine their host specificity could also be found by comparing the complete genome sequence for PCC phage species PP1 (*Podoviridae*, *Autographivirinae*, unclassified Sp6-like virus) (13), My1 (*Siphoviridae*) (14), and PM1 (*Myoviridae*) (15) and here for PPWS1, after gene functions are clarified.

### Nucleotide sequence accession number.

The entire genome sequence of *P. carotovorum* phage PPWS1 has been submitted to DDBJ under accession no. LC063634.
